# Complete Genome Sequence of Bacteriophage SM9-3Y Infecting *Serratia marcescens*

**DOI:** 10.1128/genomeA.01270-17

**Published:** 2018-01-04

**Authors:** Yuchong Hao, Hongyan Shi, Jinghua Li, Chunyan Zhao, Honglan Huang, Yanbo Sun

**Affiliations:** a Department of Pathogen Biology, College of Basic Medical Sciences, Jilin University, Changchun, People’s Republic of China

## Abstract

*Serratia marcescens* is an important opportunistic organism that causes nosocomial infections. Bacteriophage therapy has the potential to control *S. marcescens* infection. We present here the complete genome sequence of a lytic *S. marcescens* bacteriophage, SM9-3Y, which is a linear double-stranded DNA genome of 39,631 bp with 50.7% G+C content.

## GENOME ANNOUNCEMENT

*Serratia marcescens* is a Gram-negative bacillus belonging to the family *Enterobacteriaceae*. This opportunistic organism has been responsible for nosocomial infections of the respiratory tract, urinary tract, and bloodstream (1, 2). In addition, outbreaks of β-lactamase-producing *S. marcescens* in hospital settings have been frequently reported, making chemotherapy against *S. marcescens* infection difficult (3–5). Phage therapy presents a possible approach to controlling infections caused by multidrug-resistant *S. marcescens*.

Bacteriophage SM9-3Y was isolated from raw sewage collected from the First Affiliated Hospital of Jilin University, and it formed clear plaques. Bacteriophage SM9-3Y was propagated on 8 out of 25 tested clinical strains. The morphology of phage particles was measured using transmission electron microscopy. SM9-3Y showed a 48 ± 0.4-nm icosahedral capsid with a short tail and was assigned to the family *Podoviridae*. Phage DNA was sequenced at Suzhou Genewiz Biological Technology Co., Ltd. (Suzhou, China), using an Illumina MiSeq 250-bp paired-end run with a 546-bp insert library. A total of 3,095,936 reads (928,780,800 bp) were trimmed and assembled using SSPACE and GapFiller (BaseClear). Open reading frame (ORF) prediction and annotation were performed using Glimmer version 3.02 (6) and protein BLAST (7) and were subsequently confirmed using the Rapid Annotation using Subsystem Technology (RAST) server (8). tRNAscan-SE was employed to predict the presence of tRNA genes (9), but no putative genes coding for tRNAs were discovered in phage SM9-3Y.

The genome of phage SM9-3Y consists of 39,631 bp of linear double-stranded DNA (dsDNA) flanked by terminal repeats of 300 bp, with a G+C content of 50.7%. The genome of phage SM9-3Y shares high nucleotide identity (96%) with *Serratia* phage 2050H2. Of the 48 ORFs predicted, 17 encoded hypothetical proteins. The remaining 31 ORFs were categorized into 5 functional groups, that is, DNA metabolism (exonuclease, DNA-directed RNA polymerase, DNA ligase, endonuclease, DNA primase/helicase, and DNA polymerase), phage structure (head-to-tail joining protein, capsid and scaffold protein, minor capsid protein, tail tubular proteins, internal virion proteins, and tail fiber protein), packaging (DNA-packaging protein A, DNA-packaging protein B, and HNH endonuclease), lytic features (*N*-acetylmuramoyl-l-alanine amidase, holin, i-spanin, and o-spanin), and additional functions (*S*-adenosyl-l-methionine hydrolase, protein kinase, dGTP triphosphohydrolase inhibitor, bacterial RNA polymerase inhibitor, and HNS binding protein).

### Accession number(s).

The genome sequence of phage SM9-3Y has been deposited in GenBank under the accession number KX778611. The version described here is KX778611.3.

## References

[1] Merkier AK, Rodríguez MC, Togneri A, Brengi S, Osuna C, Pichel M, Cassini MH; *Serratia marcescens* Argentinean Collaborative Group, Centrón D 2013 Outbreak of a cluster with epidemic behavior due to *Serratia marcescens* after colistin administration in a hospital setting. J Clin Microbiol 51:2295–2302. doi:10.1128/JCM.03280-12. 23698525 PMC3697717

[2] Gupta N, Hocevar SN, Moulton-Meissner HA, Stevens KM, McIntyre MG, Jensen B, Kuhar DT, Noble-Wang JA, Schnatz RG, Becker SC, Kastango ES, Shehab N, Kallen AJ 2014 Outbreak of *Serratia marcescens* bloodstream infections in patients receiving parenteral nutrition prepared by a compounding pharmacy. Clin Infect Dis 59:1–8. doi:10.1093/cid/ciu218. 24729502 PMC4305132

[3] Zou L, Pan X, Wu Q, Luo Y, Liu S, Lin C, Li B, Wang X, Long M, Guo F 2011 First detection of OKP-A β-lactamase in two *Serratia marcescens* isolates in China. New Microbiol 34:371–378. 22143810

[4] Rieber H, Frontzek A, Pfeifer Y 2012 Emergence of metallo-β-lactamase GIM-1 in a clinical isolate of *Serratia marcescens*. Antimicrob Agents Chemother 56:4945–4947. doi:10.1128/AAC.00405-12. 22710114 PMC3421846

[5] Lin X, Hu Q, Zhang R, Hu Y, Xu X, Lv H 2016 Emergence of *Serratia marcescens* isolates possessing carbapenem-hydrolysing β-lactamase KPC-2 from China. J Hosp Infect 94:65–67. doi:10.1016/j.jhin.2016.04.006. 27160868

[6] Delcher AL, Harmon D, Kasif S, White O, Salzberg SL 1999 Improved microbial gene identification with Glimmer. Nucleic Acids Res 27:4636–4641. doi:10.1093/nar/27.23.4636. 10556321 PMC148753

[7] Altschul SF, Gish W, Miller W, Myers EW, Lipman DJ 1990 Basic local alignment search tool. J Mol Biol 215:403–410. doi:10.1016/S0022-2836(05)80360-2. 2231712

[8] Overbeek R, Olson R, Pusch GD, Olsen GJ, Davis JJ, Disz T, Edwards RA, Gerdes S, Parrello B, Shukla M, Vonstein V, Wattam AR, Xia F, Stevens R 2014 The SEED and the Rapid Annotation of microbial genomes using Subsystem Technology (RAST). Nucleic Acids Res 42:D206–D214. doi:10.1093/nar/gkt1226. 24293654 PMC3965101

[9] Lowe TM, Eddy SR 1997 tRNAscan-SE: a program for improved detection of transfer RNA genes in genomic sequence. Nucleic Acids Res 25:955–964. 9023104 10.1093/nar/25.5.955 PMC146525

